# Clinical and radiological outcomes in three-dimensional printing assisted revision total hip and knee arthroplasty: a systematic review

**DOI:** 10.1186/s13018-021-02646-5

**Published:** 2021-08-13

**Authors:** Rui Zhang, Jiajun Lin, Fenyong Chen, Wenge Liu, Min Chen

**Affiliations:** Department of Orthopaedics, Fujian Medical University Union Hospital, Xinquan Road No.29, Gulou District, Fuzhou, 350001 Fujian Province China

**Keywords:** Three-dimensional printing, Revision surgery, Revision total hip arthroplasty, Revision total knee arthroplasty, Systematic review

## Abstract

**Background:**

This study investigates whether three-dimensional (3D) printing-assisted revision total hip/knee arthroplasty could improve its clinical and radiological outcomes and assess the depth and breadth of research conducted on 3D printing-assisted revision total hip and knee arthroplasty.

**Methods:**

A literature search was carried out on PubMed, Web of Science, EMBASE, and the Cochrane Library. Only studies that investigated 3D printing-assisted revision total hip and knee arthroplasty were included. The author, publication year, study design, number of patients, patients’ age, the time of follow-up, surgery category, Coleman score, clinical outcomes measured, clinical outcomes conclusion, radiological outcomes measured, and radiological outcomes conclusion were extracted and analyzed.

**Results:**

Ten articles were included in our review. Three articles investigated the outcome of revision total knee arthroplasty, and seven investigated the outcome of revision total hip arthroplasty. Two papers compared a 3D printing group with a control group, and the other eight reported 3D printing treatment outcomes alone. Nine articles investigated the clinical outcomes of total hip/knee arthroplasty, and eight studied the radiological outcomes of total hip/knee arthroplasty.

**Conclusion:**

3D printing is being introduced in revision total hip and knee arthroplasty. Current literature suggests satisfactory clinical and radiological outcomes could be obtained with the assistance of 3D printing. Further long-term follow-up studies are required, particularly focusing on cost-benefit analysis, resource availability, and, importantly, the durability and biomechanics of customized prostheses using 3D printing compared to traditional techniques.

## Introduction

With the increasingly aging global population, the incidence of degenerative joint diseases, such as hip and knee osteoarthritis and necrosis of the femoral head, has increased in recent decades. Total hip arthroplasty and total knee arthroplasty are currently considered effective treatment options for end-stage joint degenerative diseases [1, 2]. The reconstruction of the artificial joint can effectively relieve pain and restore a good range of motion, greatly improving patients’ quality of daily life. As the number of primary arthroplasties has increased, complications such as periprosthetic osteolysis, aseptic loosening, periprosthetic fractures, and periprosthetic infections have increasingly been associated with the need for revision surgery. According to epidemiological studies, more than 50,000 revision total hip arthroplasty (RTHA) surgeries are performed annually in the USA [3]. Revision surgery is often more traumatic and is associated with longer operation times, more blood loss, and a greater impact on patients’ perioperative recovery. At the same time, revision surgery can cause a great mental and economic burden to patients. Orthopedic surgeons are currently working on various methods to optimize surgical procedures to achieve satisfactory outcomes while minimizing trauma.

Digital orthopedics technology represented by three-dimensional (3D) printing technology is widely used in clinical work. Patients’ computed tomography (CT) data are imported into relevant software for processing and output using a 3D printer. 3D printing technology can (1) restore the fracture model and understand the injury mechanism causing the fracture, which is conducive to better communication with patients [4]; (2) simulate the fracture reduction process in vitro and improve preoperative planning [5]; and (3) design personalized navigation or osteotomy templates to improve the accuracy of treatment [6, 7]. The application of 3D printing in primary joint arthroplasty is mainly realized by designing osteotomy guide plates [8] and customizing personalized prostheses [9, 10]. A series of clinical studies have shown that 3D printing technology can achieve excellent clinical outcomes [11–13]. With the further development of the research, orthopedic surgeons are using 3D printing technology to assist in RTHA and revision total knee arthroplasty (RTKA), especially for complex revision surgery involving bone defects.

To date, there has been no systematic review of 3D printing-assisted revision total hip and knee arthroplasty. This study aims to investigate (1) if 3D printing-assisted revision total hip and knee arthroplasty could improve clinical and radiological outcomes and (2) the depth and breadth of research conducted on 3D printing-assisted revision total hip and knee arthroplasty.

## Materials and methods

### Database and selection

**Fig. 1:**
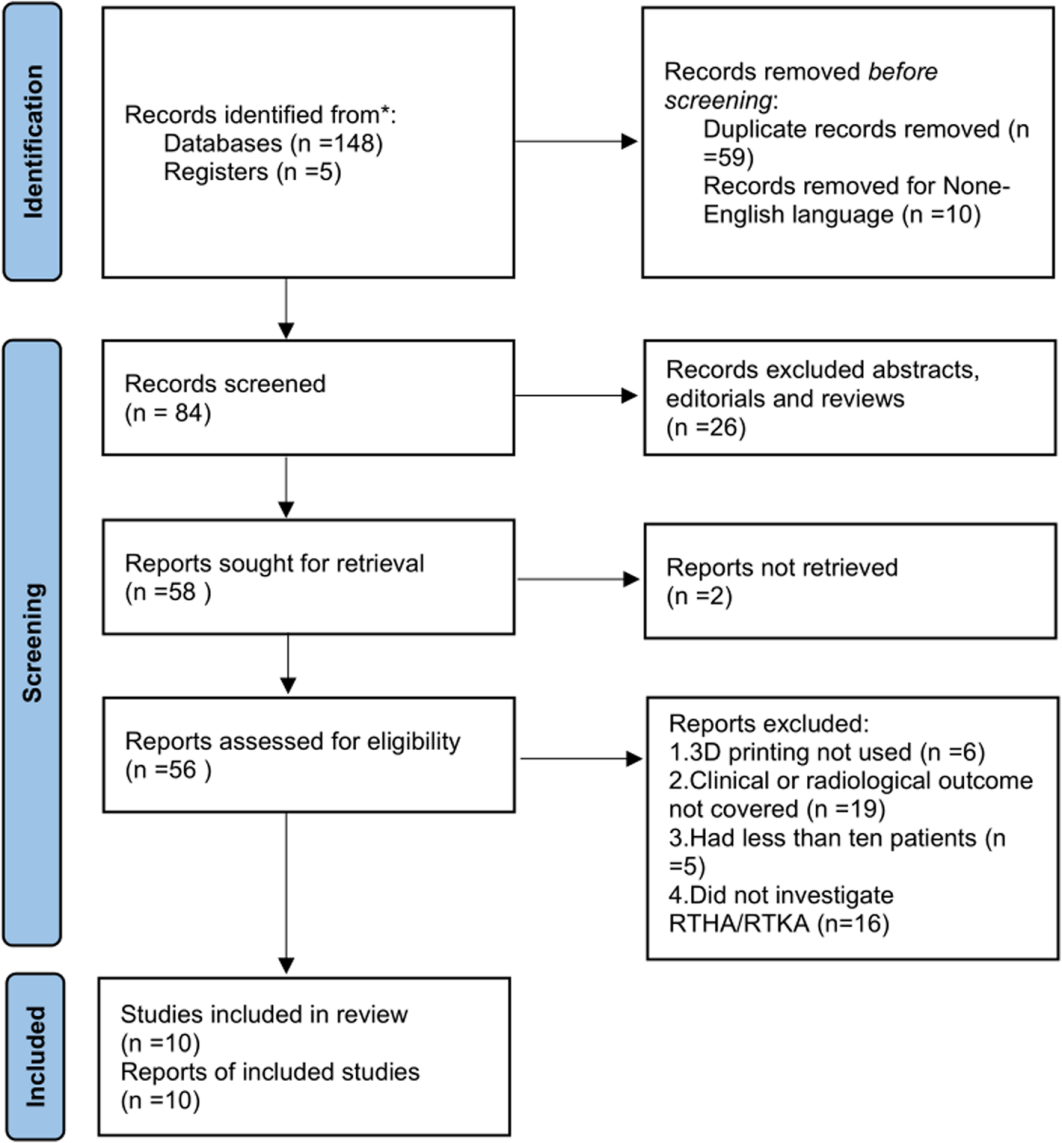
Flow diagram of the research

A literature search was carried out on PubMed, Web of Science, EMBASE, and the Cochrane Library, with retrieval performed on April 21, 2021. The following search key terms were used: “three-dimensional printing,” “3D printing,” “rapid prototyping,” “revision total hip arthroplasty,” “RTHA,” “revision total hip replacement,” “RTHR,” “revision total knee arthroplasty,” “RTKA,” “revision total knee replacement,” and “RTKR.” Only studies that investigated 3D printing-assisted revision total hip and knee arthroplasty were included. Exclusion criteria were (1) non-English language literature; (2) any abstracts, editorials, and review papers; (3) reports not retrieved or without complete data; (4) papers that did not discuss the application of 3D printing; (5) papers that did not investigate revision total hip/knee arthroplasty surgery (unicompartmental knee arthroplasty, high tibial osteotomy, and hemiarthroplasty, for example); (6) papers in which the clinical or radiological outcomes were not covered; and (7) studies including less than 10 patients. The literature search was conducted by two separate reviewers (RZ and JJL) to ensure accuracy. This systematic review was performed according to the PRISMA guidelines (http://www.prisma-statement.org/). The PRISMA checklist and flow diagram were presented in Fig. 1.

### Quality assessment

Each included paper was further reviewed by two independent reviewers (FYC and WGL) using the Coleman Methodology Score [14], widely used in orthopedic research. This scoring system is divided into two parts: part A includes study size, mean duration of follow-up, number of surgical procedures, type of study, diagnostic certainly, description of surgical procedures, and postoperative rehabilitation. Part B contains the outcome measure, outcome assessment, and selection process. The total score ranges from 0 to 100, and a higher score represents a lower influence of chance, bias, and confounding factors. The two reviewers read the full text of each study independently and gave their own scores. The average of the two reviewers’ scores was then used as a score for the study. When there is a disagreement among reviewers on the grading of a study, a third reviewer (MC) would step in, discuss, and give the final score.

### Data extraction

We extracted the following data from each included publication: author, publication year, study design, number of patients, patients’ age, the time of follow-up, surgery category, Coleman score, clinical outcomes measured, clinical outcomes conclusion, radiological outcomes measured, and radiological outcomes conclusion.

## Results

### Demographics

According to our inclusion and exclusion criteria, ten articles were included in our review, with publication years ranging from 2015 to 2021. Seven studies were retrospective clinical studies, and three were prospective clinical studies. Three articles investigated the outcome of RTKA, while seven articles investigated the outcome of RTHA. Two papers compared a 3D printing group with a control group, and the other eight papers reported 3D printing treatment outcomes alone. Besides, nine articles investigated the clinical outcomes of total hip/knee arthroplasty, and eight studied the radiological outcomes of total hip/knee arthroplasty. The average number of patients was 40.5 (range 10–139), and the average Coleman score was 60.45 (range 51.5–68) (Table 1).

**Table 1:** Summary of demographics information for each of the studies enrolled in this review

Author (Year)	Design of Study	Patients Number	Age (Yrs)	Follow-up (m)	Category	Coleman Score
Remily et al (2021) [15]	RCS	54	65(45.2-91.8)	29.9(24.0-42.0)	RTKA	52.5
Kong et al (2021) [16]	RCS	42	3D group: 65.5±11.4 Static group:67.2±10.1	3D group:18(8-28) Static group:43(30-61)	RTKA	63
Zampelis et al (2020) [17]	PCS	10	64(36-87)	12	RTHA	51.5
Tetreault et al (2020) [18]	RCS	139	66(44-88)	28.8(24-43.2)	RTKA	68
Durand et al (2020) [19]	PCS	20	66(49-90)	25.5(12-40)	RTHA	65
Wan et al (2019) [20]	PCS	42	3D group: 35.8±6.7 Control group: 34.9±5.9	12	RTHA	63.5
Li et al (2019) [21]	RCS	16	58.06 ± 8.29	27.72±12.18	RTHA	60.5
Kieser et al (2018) [22]	RCS	36	68(43-89)	38(24-108)	RTHA	66
Li et al (2016) [23]	RCS	24	65(54-79)	67(24-120)	RTHA	58
Mao et al (2015) [24]	RCS	22	60.9(38-80)	81.6±24.9	RTHA	56.5

*RCS* retrospective clinical study, *PCS* prospective clinical study, *RTKA* revision total knee arthroplasty, *RTHA* revision total hip arthroplasty

### Clinical outcomes

#### Revision total knee arthroplasty

Three articles investigated the clinical outcomes of RTKA. Remily et al. [15] and Tetreault et al. [18] reported an improved Knee Society Scores (KSS) during the perioperative period, while Kong et al. [16] indicated that the KSS scores of patients in the static group were not significantly different from the KSS score of patients in the articular group during admission. Both Remily et al. and Tetreault et al. reported good survivorship. As for complications, Remily et al. pointed out that seven surgeries were performed involving the explanation of eight cones, 11 additional knee surgeries not involving the cone were performed. Kong et al. confirmed a decreased operation time and blood loss in the 3D printing group compared to the control group. There was no significant difference between the two groups in reinfection rate, and the 3D printing group reported a better satisfaction rate (90% vs. 55%) (Table 2).

**Table 2:** Summary of clinical outcomes for each of the studies enrolled in this review

Author (Year)	Clinical Outcomes Measured	Clinical Outcome Conclusion
Remily et al (2021) [15]	KSS, complications, survivorship	Mean postoperative KSS were significantly higher when compared with preoperative KSS (80.4 vs 52.0; *p*>.001) Seven surgeries were performed involving the explantation of 8 cones, eleven additional knee surgeries not involving the cone were performed Survivorship was 98.5% when the endpoint was cone revision due to aseptic loosening. When the endpoint was considered cone revision for any reason, survivorship was 88.2%. Cone survivorship was 77.9% for any reoperation of the knee
Kong et al (2021) [16]	Operation time, blood loss, KSS, ROM, reinfection rate, satisfaction rate	The average operation time for the static group was 119 minutes (range, 75-150 minutes), whereas the average intraoperative blood loss was 439 ml (range, 250-650 ml) The average operative time of the articulating group was 98 minutes (range, 65-135minutes), whereas the average intraoperative bleeding level was 358 ml (range, 150-600 ml) The KSS score of patients in the static group was not significantly different from KSS score of patients in the articular group during admission After the second-stage revision, average ROM in the static group was 80° (70°~110°), whereas the average ROM in the articular group was 94° (80°~115°) The reinfection rate in static group was (1/22), whereas in articular group was (1/20), and there was no significant difference between two group (*p*=0.95) After the final revision surgery, 55 % (12/22) of patients in the static group were satisfied with overall treatment, whereas 90% (18/20) of patients in the articular group were satisfied with overall treatment
Tetreault et al (2020) [18]	KSS, survivorship, complications	The mean KSS improved from 50 (0 to 94) preoperatively to 87 (72 to 94) at the most recent follow-up (*p* < 0.001) At two years, survivorship free of cone revision for aseptic loosening was 100% and free of any cone revision was 98% (95% CI = 95% to 100%). Two- year survivorship free of any revision was 90% (95% CI = 86% to 96%), and free of any reoperation was 83% (95% CI = 77% to 90%) Two patients had three intraoperative complications related to cone placement. There were two distal femoral fractures with implant removal and one partial patellar tendon avulsion during exposure. There were two instances of recurrent PJI and one of partial wound dehiscence managed non-operatively. additionally, one patient had a nonfatal pulmonary embolism postoperatively
Durand et al (2020) [19]	OHS, post-operative walking status, complications	Mean OHS at latest follow up was 32.4 (S.D 10.4) There were eight patients need crutches, one patient need wheelchair, five patients were walking independent, five patients were unilateral walking stick, and one patient need frame One patient occurred periprosthetic fracture, and required revision
Wan et al (2019) [20]	HSS, VAS, SF-36	There was no significant difference in HSS between the two groups before operation (*p*>0.05). At 3, 6 and 12 months after operation, HSS of the observation group were significantly higher than those of the control group (*p*<0.05) There was no significant difference in VAS score between the groups before operation (*p*>0.05). VAS scores of the observation group were significantly lower than those of the control group at 3, 6 and 12 months after operation (*p*<0.05) There was no significant difference in SF-36 scores between the two groups before treatment (*p*>0.05). The SF-36 scores of the observation group were significantly higher than those of the control group at 3, 6 and 12 months after treatment (*p*<0.05)
Li et al (2019) [21]	Operation time, blood loss, blood transfusion, HSS, complications	The mean operation time was 254 ± 91 min The blood loss was 891 ± 423 ml The mean blood transfusion was 860 ± 400 ml The mean HHS was significantly improved from 27.50 ± 6.54 preoperatively to 80.94 ± 5.19 at final follow-up (*p*<0.001) Two cases had intraoperative periprosthetic femoral fracture
Kieser et al (2018) [22]	OHS, HSS, WOMAC, complications	3 patients reported no disability (OHS 60), 15 excellent outcomes (OHS >50), 11 a good outcome (OHS 40-50) and 6 a fair outcome (OHS 30-40) 2 patients were reported to be doing poorly (OHS 29 and 27 respectively) at >2 years follow-up. 1 patient had early implant migration with subsequent stabilization. 2 patients had radiographs concerning for failure of osteointegration. 1 patient had recurrent dislocations.
Li et al (2016) [23]	HSS, complications	HSS scores improved from a mean of 36 before surgery (SD, 8; range, 20–49) to 82 at latest follow-up (SD,18; range, 60–96; *p*<0.001) Infections developed in two hips, and one dislocation case
Mao et al (2015) [24]	HSS, complications, survivorship	The mean HSS score improved from 39.6 (range, 12–60) pre-operatively to 80.9 (range, 53–93) at the final follow-up (*p*<0.01) One patient who had an intra-operative rupture of the superior acetabular artery, and two patients experienced dislocation The survival of the cage with revision for any reason was 91.30 % (95 % CI 58.10–73.95) and with aseptic loosening as the endpoint was 95.66 % (95 % CI 63.10–74.81)

*KSS* knee society scores, *ROM* range of motion, *OHS* Oxford Hip scores, *VAS* visual analogue scale, *HSS* Harris Hip Scores, *WOMAC* Western Ontario and McMaster Universities Arthritis Index, *SF*-36, Health Survey Scale-36, *SD* standard deviation, *CI* confidence level

#### Revision total hip arthroplasty

Six articles reported the clinical outcomes of RTHA. Of these six articles, five articles used Harris Hip Scores (HSS) to evaluate hip function after revision surgery and reported significant improvement from the preoperative period to the final follow-up. Durand et al. [19] also investigated the postoperative walking status. He found eight patients in need of crutches, one in need of a wheelchair, five patients walked independently, five required a unilateral walking stick, and one patient needed a walking frame. Wan et al. [20] indicated that visual analog scale scores of the observation group were significantly lower than those of the control group postoperatively. In comparison, the Health Survey Scale-36 (SF-36) scores of the observation group were significantly higher than those of the control group after treatment. Mao et al. [24] reported good cage survivorship using a 3D printing technique. Periprosthetic fracture, infection, dislocation, and intra-operative arterial rupture were seen as common complications listed in included publications (Table 2).

### Radiological outcomes

#### Revision total knee arthroplasty

Only one article studied the radiological outcome of RTKA. The author used the Knee Society Radiological Evaluation Criteria as an evaluation tool. He found that most cases’ radiographs showed evidence of osseointegration with reactive osseous trabeculation at the interface. Few cases considered radiological failures with evidence of loosening (Table 3).

**Table 3:** Summary of radiological outcome for each of the studies enrolled in this review.

Author (Year)	Radiological Outcomes Measured	Radiological Outcome Conclusion
Zampelis et al (2020) [17]	Inclination, anteversion, rotation, COR	There was a median deviation in postoperative position versus planned in inclination of 3.6° (IQR 1.0 to 5.4) There was a median deviation in postoperative position versus planned in anteversion of –2.8° (IQR –7.5 to 1.2) There was a median deviation in postoperative position versus planned in rotation of –1.2° (IQR –3.3 to 0.0) The median deviation in position of COR was –0.5 mm (IQR –2.9 to 0.7) in the AP plane, –0.6 mm (IQR –1.8 to –0.1) in the ML plane, and 1.1 mm (IQR –1.6 to 2.8) in the SI plane
Tetreault et al (2020) [18]	Knee society radiological evaluation criteria	Follow-up radiographs showed evidence of osseointegration with reactive osseous trabeculation at the interface in 98% (119/122) of unrevised cases Three unrevised femoral-sided cones were considered radiological failures with evidence of loosening All four instances of cone loosening occurred in patients with type 2B or 3 defects in the presence of a hinged implant
Durand et al (2020) [19]	COR, component rotation, inclination and version cup angles	All components (100%) were positioned within 10mm of planned COR (in the three planes). Eighteen (95%) components were not rotated by more than 10° compared to plan. Eleven (58%) components were positioned within 5° of planned cup angle (inclination and version) The mean difference, between planned and achieved, COR was -0.1 mm (95% CI -8.7, 8.6) in the AP plane, -1.4mm (95% CI-7.6, 4.8) in the SI plane and 0.1 mm (95% CI-9.4, 9.5) in the ML plane The mean deviation of component rotation from planned was 2.2° (-6.4, 10.8) Planned inclination had a mean of 40.3°(95% CI 29.2,51.5) and 40.5°(95% CI 26.6,54.4) postoperatively, the difference between the planned and postoperative inclination was 0.2°(95% CI -10.2 to 10.7) The mean planned version was 14.2° (95% CI -3.5, 31.9), version post-operatively was 17.0° (95% CI -0.7, 33.3) with a mean difference between planned and postoperative version of 2.8° (95% CI -10.5, 16.1)
Wan et al (2019) [20]	The DeLee and Charnley zoning method, the bone growth evaluation criteria of the Anderson Orthopaedic Institute	There was no change in displacement and abduction angle in the observation group None of the patients showed a bright line at the last follow up Revision failed in one patient, 18 patients had no loosening at 6 months after surgery and 18 patients had continuous trabecular passage at the junction of prosthesis and host bone
Li et al (2019) [21]	Acetabular cup anteversion angle, acetabular cup abduction angle, COR, safe zone	11 of 18 (61.1%) patients were positioned within the safe zone Percentage of outliers was corrected from 77.78% (14/18) preoperatively to 38.89% (7/18) postoperatively, with statistical significance (*p* = 0.040) Ratio of vertical position of COR in surgical site/contralateral site was corrected from 1.15±0.19 to 1.09 ± 0.20 postoperatively (*p* = 0.185) Ratio of horizontal position of COR in surgical site/contralateral site was changed from 0.97 ± 0.21 to 1.00 ± 0.18 postoperatively (*p* = 0.193) The mean planned cup anteversion value did not differ from the postoperative value (−1.39 ± 4.1; *p*= 0.168), and a strong correlation was found (*r* = 0.894; *p* < 0.001) There was deviation between the mean planned abduction and the postoperative value (2.24 ± 3.02; *p*=0.006), but a strong correlation between these two values was found (*r*=0.921, *p*< 0.001)
Kieser et al (2018) [22]	Moore criteria, migration of hip center, asymmetrical wear	All other patients had stable implants with evidence of osteointegration. No patients experienced radiographically apparent premature liner wear. The average change in Brooker score, at 2-years, was 0.3 (range 0-3, p=0.090).
Li et al (2016) [23]	Inclination of the cage, COR, component migration	Individualized custom cages resulted in generally reliable restoration of the hip center The mean vertical distance was 25 mm (SD, 5 mm; range, 19–40 mm) on the revised side and 24 mm (SD, 5 mm; range, 18–40 mm) on the contralateral side (*p*=0.265) The mean horizontal distance was 106 mm (SD, 9 mm; range, 90–119 mm) on the revised side and 109 mm (SD, 9 mm; range, 94–123 mm) on the other side (*p*=0.75) Radiographic analysis showed the mean inclination of the cage was 46° (SD, 6°; range, 38°–58°) No definite migration of any of the acetabular cups was observed
Mao et al (2015) [24]	The DeLee and Charnley zoning method, the stability of the cage	22 of the 23 cages (including the re-revision case) were considered stable and without migration based on the radiographic data The overall incidence of radiolucent lines was 13% (three hips) In all cases, the radiolucency was partial and nonprogressive, and the lines were<2mm in width Incorporation of the graft complete in 19 hips. No screw fractures were observed

*COR* center of rotation, *IQR* interquartile range, *AP* anteroposterior, *ML* mediolateral, *SI* superoinferior, *CI* confidence level

#### Revision total hip arthroplasty

Four of the seven enrolled articles concerning RTHA used the center of rotation (COR) as an indicator to evaluating radiological outcomes. These studies reported a reliable restoration of the hip center within the tolerance for error. In addition, inclination, anteversion, rotation, version cup angles, and acetabular cup abduction angle are also listed as measured radiological outcomes. Two studies used the DeLee and Charnley zoning method to evaluating radiological outcomes and indicated that revision implants were considered stable and without migration based on the radiographic data. Wan et al. [20] utilized the bone growth evaluation criteria of the Anderson Orthopaedic Institute as an evaluation index and concluded that there was no change in displacement and abduction angle in the observation group. Meanwhile, none of the patients showed a bright line at their final follow-up (Table 3).

## Discussion

This article reviews the current use of 3D printing in revision total hip and knee arthroplasty, showing applications ranging from industrial to the production of custom-made prostheses or assisting in complex revision surgery. To our knowledge, this is the first paper specifically reviewing the applications of 3D printing technology in revision total hip and knee arthroplasty.

With the continuous innovation of artificial joint prosthesis materials and design, the progress of surgical techniques and methods, increasing attention to preoperative preparation and postoperative rehabilitation, and the standard use of antibiotics during and after surgery, the incidence of related joint arthroplasty-complications has been gradually reduced. The survivorship of prostheses is also becoming much longer. However, it is also due to the development of arthroplasty, the increase in the absolute number of procedures, and the increase in human longevity that the number of patients undergoing revision surgery after joint arthroplasty is increasing. While revision surgery does provide excellent clinical benefits and patient satisfaction, the procedure is technically challenging and often results in poor outcomes compared to primary arthroplasty. Revision surgery is believed to be associated with higher postoperative complications (such as re-aseptic loosening, periprosthetic fractures, infections, dislocation, and more difficult postoperative recovery). According to reports by Deng et al., Yoshimoto et al., and Ong et al., the probability of femoral fracture around the prosthesis after the revision was three times higher than that of total hip arthroplasty [25], and the re-dislocation rate after the revision was 18.2% [26], which was significantly higher than the initial arthroplasty. The incidence of infection after primary arthroplasty is about 0.57%, and the reinfection rate after prosthesis infection and revision increase to 5% [27]. This leads to the conclusion that revision surgery sometimes has significantly worse outcomes than those with primary arthroplasty.

Seven of ten articles reported perioperative complications [15, 18, 19, 21–24]. For RTKA surgery, perioperative complications mainly concerned aseptic loosening [15], periprosthetic fracture [18], and periprosthetic joint infection [18]. In RTHA surgery, complications focus on periprosthetic fracture [19, 21], recurrent dislocations [22–24], periprosthetic joint infection [23], and aseptic loosening [24]. However, both these studies reported lower complications rate which indicated that 3D printing technology exist some advantages compared with traditional surgery in reducing complication rates.

The goals of RTHA are to achieving a stable hip, restoring the hip’s COR, restoring the length of the limb, and obtaining optimal initial and long-term fixation outcomes [28]. Successful acetabular revision requires close contact between the acetabular prosthesis and the surface of the acetabular bone, stable fixation of the prosthesis, and minimization of micromotion between the prosthesis and the bone surface to ensure long-term bone ingrowth of the surface of the prosthesis, which results in a stable mechanical structure that allows stress to be distributed around the prosthesis [29]. In addition, this restores the typical biomechanical environment of the hip joint by reconstructing the hip anatomic center. Four studies [17, 19, 21, 23] used COR as an indicator to evaluate radiological outcomes. These studies reported a reliable restoration of the hip’s center with the assistance of 3D printing. The favorable reconstructed acetabular position (anteversion angle and abduction angle) can reduce the risk of postoperative dislocation. Three studies [17, 19, 21] investigated radiological outcomes according to the anteversion angle, abduction angle, and other indicators. These publications both reported well reconstructed acetabular cup positions within the tolerance for error. The HSS system provides a detailed reference for clinicians to evaluate patients’ daily living ability, gait, and hip range of motion. Five articles [20–24] used HSS to evaluate hip function after revision surgery and reported significant improvement from the preoperative period to final follow-up. As for femoral stem revision, by restoring the hip joint’s biomechanical structure, the axial and rotational stability of the femoral stem prosthesis was achieved. In this review, no studies reported femoral stem revision. KSS is a systematic score system that comprises daily living ability, knee range of motion, and other indicators. Three articles [15, 16, 18] both indicated an improved KSS during the perioperative period.

RTKA is technically more challenging than the primary replacement because the combined bone defect makes prosthesis fixation and force line maintenance more complex [30]. The objectives of RTKA include correction of lower limb alignment, correction of prosthesis position, maintenance of soft tissue balance in the extension and flexion positions, restoration of the normal transverse joint position, correction of the patella’s motion trajectory, and increasing of range of motion to meet the needs of daily life [31]. Tetreault et al. [18] used the knee society radiological evaluation criteria reported that follow-up radiographs showed evidence of osseointegration with reactive osseous trabeculation at the interface in 98% (119/122) of unrevised cases. This kind of custom-made prosthesis is easy to repair bone defects and regain soft tissue balance.

3D printing integrates modern digital capture technology, computer-aided design (CAD), numerical control technology, laser or electron beam technology, and the latest achievements in materials science [32]. There have been significant recent advances in 3D printing technology and its associated software. In the medical field, a combination of 3D printing and CT scanning equipment will allow the capture of a digital grid model in a biological form to 3D print a corresponding physical object, reverse engineered to be the same shape and internal structure as the biological 3D object [33]. Therefore, the application of 3D printing technology in medical reconstruction has created a new field, namely digital medicine.

Kong et al. [16] investigated the application of a 3D printing-assisted articulating spacer in two-stage revision surgery for periprosthetic infection after total knee arthroplasty and concluded that the 3D printed articular spacer provides a satisfactory range of motion, postoperative rehabilitation, and results in low reinfection and complication rates. The remaining nine studies were conducted on 3D printed custom prostheses. During revision surgery, there is a high probability that the morphology of the prosthesis and the surgical site are poorly matched. In such cases, the prosthesis cannot achieve the best match with the residual structure; thus, the optimal reconstruction of the affected limb function cannot be achieved after surgery [34]. Surgeons often have two options for dealing with this problem; the mainstream treatment method is to modify the adjacent bone structure based on the conventional prosthesis to achieve a match between the implant and the adjacent bone structure. The other option involves designing and manufacturing implants that perfectly match the patient’s local anatomy and can withstand its stress characteristics [35]. The former tends to result in insufficient strength of the local support structure, uneven stress conduction, and subsequent unsatisfactory bone reconstruction, increasing the risk of systemic complications of fat embolism and causing severe consequences. Theoretically, the latter is more suitable for patients than the former because it causes less damage to local bony structures.

Besides, 3D printed customized implants, after optimizing the mechanical structure, that adopt a nanoporous microstructure were designed and manufactured using materials with high cell affinity. This encourages osteoblasts to proliferate in the artificial joint rather than on the implant surface, resulting in a greater spread than that with traditional joint replacement, which produces a larger contact area and greater stress resistance [36]. The improvement of the stress resistance of the artificial joint is bound to increase its long-term survival rate. In our literature review process, we found that articles reported satisfactory revision prosthesis survivorship with a low incidence of postoperative complications.

A major strength of the present systematic review is the strict adherence to the PRISMA protocol and the use of accurate inclusion and exclusion criteria, which made our study reliable, since all the most up-to-date scientific evidence about the topic was meticulously examined.

Our systematic review has some limitations. Firstly, only specific studies (English language original articles) were included in this review, which may have led us to overlook other high-quality literature in other languages in the field of 3D printing assisted revision total hip/knee arthroplasty. Secondly, although all enrolled papers reported clinical research, there were a small number of excluded control studies with an insufficient patient’s number of patients for analysis. This may be because of the costs of 3D printing and the recruitment of patients. Thirdly, the follow-up duration varied between studies which may produce biased results.

Currently, there are relatively few clinical studies on 3D printing-assisted RTHA and RTKA. According to our inclusion and exclusion criteria, only 10 studies have been included. Even fewer studies both investigated clinical and radiological outcomes [18–24], and the evaluation indicators were also lack of uniformity.

The application of 3D printing technology in orthopedic clinical diagnosis and treatment has made continuous progress, but 3D printing technology in revision total hip and knee arthroplasty is still in the initial stage, and many problems need to be solved in the future. For instance, what are the biocompatibility and long-term bone ingrowth effects of the revision prosthesis? Also, larger, multicenter randomized controlled clinical trials will be needed in the future to investigate the effects of 3D printing in revision total hip and knee surgery.

## Conclusion

3D printing is being introduced in revision total hip and knee arthroplasty. At present, the clinical application is mainly customized personalized prostheses and articulating spacers, and relevant research has pointed out its satisfactory clinical and radiological outcomes. Orthopedic surgeons should be familiar with its potential effects, advantages, and disadvantages. Further long-term follow-up studies are required, particularly focusing on cost-benefit analysis, resource availability, and importantly, the durability and biomechanics of personalized prostheses customized using 3D printing compared to traditional techniques.

## Data Availability

The datasets generated and/or analyzed during the current study are available in the PubMed, Web of Science, EMBASE, and the Cochrane Library.

## Abbreviations

RTHA: Revision total hip arthroplasty
RTHR: Revision total hip replacement
RTKA: Revision total knee arthroplasty
RTKR: Revision total knee arthroplasty
3D: Three-dimensional
CT: Computed tomography
RCS: Retrospective clinical study
PCS: Prospective clinical study
KSS: Knee Society Scores
ROM: Range of motion
OHS: Oxford Hip scores
VAS: Visual analog scale
HSS: Harris Hip Scores
WOMAC: Western Ontario and McMaster Universities Arthritis Index
SF-36: Health Survey Scale-36
SD: Standard deviation
CI: Confidence level
COR: Center of rotation
IQR: Interquartile range
AP: Anteroposterior
ML: Mediolateral
SI: Superoinferior
